# A systematic review of barriers to data sharing in public health

**DOI:** 10.1186/1471-2458-14-1144

**Published:** 2014-11-05

**Authors:** Willem G van Panhuis, Proma Paul, Claudia Emerson, John Grefenstette, Richard Wilder, Abraham J Herbst, David Heymann, Donald S Burke

**Affiliations:** University of Pittsburgh Graduate School of Public Health, DeSoto street 130, 703 Parran Hall, Pittsburgh, PA 15261 USA; Sandra Rotman Centre, University Health Network & University of Toronto, Toronto, Canada; Bill & Melinda Gates Foundation, Seattle, USA; INDEPTH Network, Accra, Ghana; The Africa Centre for Health and Population Studies, UKZN, Somkhele, South Africa; Chatham House London, London, UK

**Keywords:** Data sharing, Public health, Surveillance

## Abstract

**Background:**

In the current information age, the use of data has become essential for decision making in public health at the local, national, and global level. Despite a global commitment to the use and sharing of public health data, this can be challenging in reality. No systematic framework or global operational guidelines have been created for data sharing in public health. Barriers at different levels have limited data sharing but have only been anecdotally discussed or in the context of specific case studies. Incomplete systematic evidence on the scope and variety of these barriers has limited opportunities to maximize the value and use of public health data for science and policy.

**Methods:**

We conducted a systematic literature review of potential barriers to public health data sharing. Documents that described barriers to sharing of routinely collected public health data were eligible for inclusion and reviewed independently by a team of experts. We grouped identified barriers in a taxonomy for a focused international dialogue on solutions.

**Results:**

Twenty potential barriers were identified and classified in six categories: technical, motivational, economic, political, legal and ethical. The first three categories are deeply rooted in well-known challenges of health information systems for which structural solutions have yet to be found; the last three have solutions that lie in an international dialogue aimed at generating consensus on policies and instruments for data sharing.

**Conclusions:**

The simultaneous effect of multiple interacting barriers ranging from technical to intangible issues has greatly complicated advances in public health data sharing. A systematic framework of barriers to data sharing in public health will be essential to accelerate the use of valuable information for the global good.

**Electronic supplementary material:**

The online version of this article (doi:10.1186/1471-2458-14-1144) contains supplementary material, which is available to authorized users.

## Background

Public health decision making has become increasingly complex and the use of data has become essential in this information age [1]. At the local level, data are used to monitor population health and to target interventions; at the national level, data are used for resource allocation, prioritization, and planning; and at the global level for estimates on the global burden of disease, to measure progress in health and development, and to contain emerging global health threats [2–7]. In addition to their primary use by public health agencies, routinely collected public health data have become valuable for secondary use such as academic research and technology development. Recently, global health and funding agencies have made appeals for greater availability and access to granular public health data [2, 8] and have developed principles for data sharing in global health [8, 9].

Benefits of data sharing have been widely recognized – transparency and cooperation, reproducibility of research, cost-efficiency and preventing redundancies, acceleration of discovery and innovation, and saving lives through more efficient and effective public health programs [5, 10–12]. Despite a growing global commitment to the use and sharing of public health data, this can be challenging in reality. For example the global polio eradication initiative (GPEI) could benefit from more widely available genetic sequence data to reconstruct chains of transmission, and estimates made by the global burden of disease project (GBD) and the Millennium Development Goals (MDG) would be more accurate if better quality data would be available from parts of the world [2, 5, 6, 13, 14]. Even at the local level, the efficient use and sharing of data among different agencies can be a challenge.

The field of public health is highly interdisciplinary and includes a wide range of data sources that is always evolving in size and complexity. Much data is derived directly from populations monitored by health agencies such as from clinical records and demographic and survey data. In addition, many auxiliary data sources are used to measure determinants of health such as environmental, climate, social behavior, transport, and other types of data [4]. Although overlap exists across types of data, this paper will focus on routinely collected population derived public health data such as disease surveillance data, intervention coverage data, vital statistics and cause specific mortality data. These represent some of the most widely collected, but also some of the most underused data sources in public health science and policy.

A global policy framework or operational guidelines for data sharing in public health have not yet been developed for most types of data. For example census and survey data are increasingly shared through centralized platforms such as the International Household Survey Network (IHSN) [15] or the International Public Use Microdata Series [16], but progress in sharing of disease surveillance data or cause specific mortality data has been slow. Many potential and real barriers to sharing of public health data have been recognized such as privacy issues or legal constraints but so far have only been anecdotally discussed or presented in the context of specific examples and case studies. This has led to disjointed and incomplete evidence on the scope and variety of challenges that currently limit data sharing. Unless these barriers are better understood, solutions may remain ineffective. We conducted a systematic literature review of potential barriers to data sharing and used this evidence to group these barriers in a taxonomy that can be used as a framework to facilitate an international dialogue on solutions and instruments to advance data sharing for better population health.

## Methods

We conducted a systematic review according to PRISMA guidelines [17] to identify documents that reported on barriers to data sharing in public health [see Additional file 1]. We defined public health data as data that were primarily collected by public health agencies for routine purposes such as disease surveillance or program monitoring without primary intention of research [4, 18–20]. Barriers were defined as obstacles that could impede or delay data sharing or that could limit the efficiency of data sharing in public health. Studies describing barriers on clinical (patient oriented) or research data were excluded. The protocol for this review has been provided as supporting information [see Additional file 2].

We searched the MEDLINE database in August 2013 for original English-language research articles using two different queries. The first query was [“public health” OR “world health”] AND [“data sharing” OR “data access” OR “open access” OR “dissemination” OR “sharing practices”] AND [“barriers” OR “challenges”]. The second query used the following combination of key words: [“population surveillance” OR “health statistics” or “vital statistics” or “civil registry” or “health data”] AND [“data sharing” OR “data access” OR “open access” OR “dissemination” OR “sharing practices”] AND [“barriers” OR “challenges”]. Additional documentation was identified through the bibliographies of indexed papers and websites of major international agencies such as the WHO, the US Centers for Disease Control (CDC), and the Wellcome Trust.

**Figure 1:**
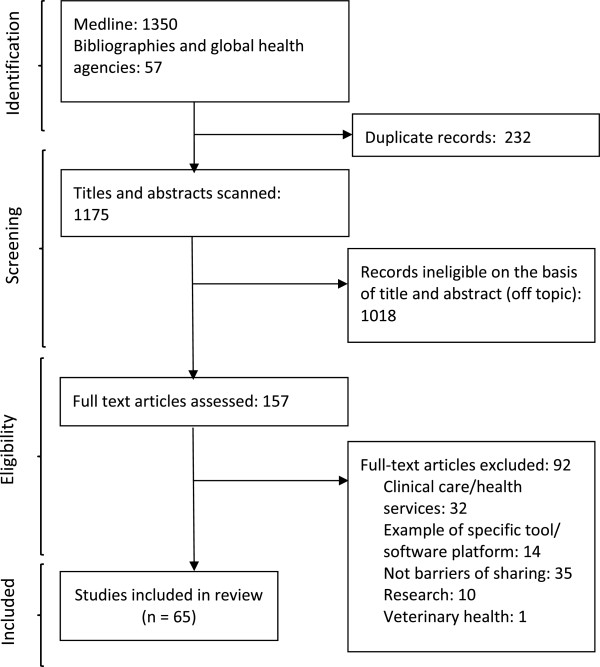
**Systematic selection of studies on barriers to public health data sharing from the peer-reviewed and grey literature.**

We identified 1350 articles in MEDLINE and 57 articles from bibliographies and agency websites (Figure 1). Of these, 232 were duplicate articles and 1018 were excluded based on title and abstract review. We reviewed the full text of the remaining 157 papers. Ninety-two studies were excluded because they focused on clinical data (32), described tools for data sharing but no barriers (14), described data sharing but no barriers (35), focused on research data instead of public health data (10) or focused on animal health (1). Sixty-five studies were finally included in this review. All of these studies were initially read independently by two investigators and an initial list of barrier descriptions was extracted. This list was reviewed by domain experts among the authors and classified into preliminary categories. Experts then grouped and generalized barrier descriptions within their categories. Iteratively, a modified list of barriers was proposed and compared to the original barrier descriptions to preserve the intent of the source documents. A final taxonomy and description of barriers emerged from a series of group discussions. For each barrier, we also categorized available evidence to identify knowledge gaps. We classified studies published in peer-reviewed papers or not and presenting empirically derived evidence (through original data collection such as interviews, focus groups, etc.) or not.

## Results

We identified 20 unique real or potential barriers to data sharing in public health and classified these in a taxonomy of six categories: technical, motivational, economic, political, legal, and ethical barriers (Table 1). These barriers and categories describe a landscape of challenges that is highly dynamic, interconnected, and hierarchical. Although most evidence (68%) was published in peer-reviewed sources, less than a quarter (22%) of all the documents reviewed was based on empirically derived evidence, indicating that a large volume of published expert knowledge has not yet been translated into scientific evidence.

**Table 1:** **Evidence for barriers to sharing of routinely collected public health data**

Category	Barrier	Peer-reviewed		Non peer-reviewed
		Empirical data	Non-empirical*	
**Technical**	1. Data not collected	[6, 21, 24, 31]	[2, 4, 7, 18, 22, 14, 26–28, 30]	[3, 23, 25]
	2. Data not preserved		[33]	[3, 32, 34, 35]
	3. Data not found		[45]	[3, 34]
	4. Language barrier			[36]
	5. Restrictive data format		[40]	[3, 34, 36–39, 41]
	6. Technical solutions not available		[42]	[37]
	7. Lack of metadata and standards	[21, 24, 43]	[40, 44, 45]	[1, 35–37, 39, 41, 46]
**Motivational**	8. No incentives		[27, 45, 49]	[35]
	9. Opportunity cost	[51, 52]	[13, 33, 50, 53]	[35]
	10. Possible criticism		[33]	[32]
	11. Disagreement on data use	[21]	[49]	
**Economic**	12. Possible economic damage		[7, 26, 27, 30]	[55]
	13. Lack of resources	[56, 21]	[13, 27, 28, 30, 42, 53, 57]	[3, 23, 34–36, 39, 37]
**Political**	14. Lack of trust	[19, 59, 60]	[33, 61]	[34–37]
	15. Restrictive policies		[30]	
	16. Lack of guidelines		[45, 62, 65]	[37, 41, 63, 64]
**Legal**	17. Ownership and copyright		[62, 65, 66, 69]	[37, 63, 64, 67]
	18. Protection of privacy	[12, 19, 59, 73, 75]	[44, 57, 62, 66, 72, 74]	[36, 37, 64, 67, 68, 70, 71]
**Ethical**	19. Lack of proportionality			[76]
	20. Lack of reciprocity	[51, 52]	[50, 77, 78]	
Number of unique documents (% of total)		14 (21.5%)	30 (46.2%)	21 (32.3%)

*No or little original data presented.

### Technical barriers

These barriers for the most part are well understood as part of resilient challenges in health information system capacity and continue to form a major obstacle to the availability and use of public health data. Solutions to these barriers have been identified but sustainable implementation and political/financial commitment have been limited.

1. *Data not collected.* As long as severe limitations persist in public health data collection, data sharing will not be considered a priority. The WHO Health Metrics Network, the CDC/USAID Data for Decision Making project (DDM) and other agencies have found significant gaps in public health data systems, in particular in low- and middle income countries [2–4, 21–25]. Disease surveillance systems in many countries cannot meet standards set by the 2005 International Health Regulations [7, 25–30]. Civil registration systems in many countries are lacking as well [2, 6, 14, 18, 22, 24, 31].
2. *Data not preserved or 3, cannot be found.* Public health data are often collected for short-term purposes such as outbreak detection. Data preservation or archiving is often not prioritized, especially in situations of limited capacity and resources [3, 18, 32–35]. Even if data have been preserved, data retrieval systems may be lacking. This is amplified by relocation of offices, staff turnover, physical damage to paper or electronic files, computer viruses, computer theft, etc. [34].
3. *Language barrier.* Routinely collected public health data are often recorded in local languages, limiting the possibility to integrate and use such data together with other data sets, particularly in an international context [36].
4. *Restrictive data format.* Despite major advances in computational resources in public health, a large volume of public health data such as disease surveillance data and administrative data continue to be collected and preserved in hardcopy paper format or in electronic format that may be antiquated or incompatible with modern software systems [3, 34, 36–41].
5. *Technical solutions not available.* Technical software solutions to collect, harmonize (transformation and recoding to enhance inter-operability), integrate (combining harmonized datasets), and share complex and heterogeneous data have been developed in the private or research sector, but have not become widely available to public health agencies [37, 42].
6. *Lack of metadata and standards.* Oftentimes, metadata that describe data content, origin, methods, etc. are lacking for public health data and standards for data format, variables, and metadata are insufficiently used, limiting secondary data use and inter-operability [1, 21, 24, 35–37, 39–41, 43–46]. Some advances have been made through the development of the International Classification of Diseases (ICD) [47], the Data Documentation Initiative (DDI) and the Standard Data and Metadata eXchange (SDMX) [48]. These standards are not always used efficiently however. For example, between 1950 and 2010, up to 20% of deaths in certain countries were attributed to ill-defined ICD codes [24].

### Motivational barriers

These include barriers based on personal or institutional motivations and beliefs that limit data sharing. Solutions for this group of barriers lie in building trust or developing transparent legal agreements.

8. *No incentives.* Data sharing requires time and resources that are chronically lacking in public health settings [27, 35, 37]. Personal and institutional incentives are often required to prioritize data sharing over other pressing duties [45, 49], particularly if the benefit of data sharing is delayed and uncertain (e.g. possibly more efficient disease control programs) instead of immediately relevant to data providers (e.g. scientific credit or training).
9. *Opportunity cost.* Public health officers who have invested time and effort in data collection could anticipate that scientific credit or other opportunities may be lost if data recipients with greater capacity for analysis could gain the majority of credit [13, 33, 35, 50–52]. This is a particular challenge in low resource settings [50, 53].
10. *Possible criticism.* Data providers could be discredited by errors found during secondary use of their data and disease control efforts may be criticized if data would reveal continued disease occurrence [32, 33]. In the worst case, data sharing could reveal data fabrication or manipulation. For example, studies have shown over-reporting of vaccine coverage by country statistics compared to independent surveys after introduction of GAVI incentive funding for vaccination programs [54].
11. *Disagreement on data use.* Data providers may disagree with the intended secondary use of their data or may consider their data inappropriate for a certain use [49].

### Economic barriers

These barriers concern the potential and real cost of data sharing and solutions depend on the recognition of data value and on sustainable financing mechanisms.

12. *Possible economic damage*. Data sharing in public health is challenged by the economic damage that this may cause to data providers. Public sharing of disease outbreak data, for example, can result in economic damage due to reduced tourism and trade [7, 26, 27, 30, 55]. The global SARS outbreak led to estimated economic losses of 50 billion USD between 1998 and 2004 and Foot & Mouth Disease in the UK resulted in losses of 30 billion USD between 1998 and 2003 [55]. The possibility of such significant economic implications due to (over) reactive market forces could cause great reluctance among health agencies to rapidly release disease data.
13. *Lack of resources.* The process of data sharing requires human and technical resources for data preparation, annotation, communication with recipients, computer equipment, internet connectivity, etc. [3, 21, 34, 35, 42, 53]. These resources are frequently lacking in public sector agencies under economic pressure or in low income settings [3, 13, 21, 23, 25, 28, 30, 34, 36, 37, 56, 57].

### Political barriers

These are fundamental structural barriers embedded in the public health governance system that are grounded in a political or socio-cultural context. Solutions for these barriers are not clear-cut and will require global and national processes to build consensus and political will.

14. *Lack of trust.* Trust between a data provider and user greatly enables data sharing [37]. In the absence of trust, providers could anticipate potential misinterpretation, misuse or intentional abuse of the data [19, 33–36, 58, 59]. For example the Indonesian government refused to share H5N1 influenza samples with the international community during the 2007 pandemic due to lack of trust on the potential use of these samples for financial gain [60]. Legal arrangements were required in the absence of a trust relationship which led to the development of the Pandemic Influenza Preparedness Framework [61].
15. *Restrictive policies.* Agencies may have developed official policy guidelines that restrict data sharing, resulting from various possible underlying factors such as a general sense of distrust, negative prior experiences, or other factors [30].
16. *Lack of guidelines.* Frequently, official guidelines on data sharing simply do not exist, are unclear or inconsistent [37, 41, 45, 62, 63]. The balance between making data accessible, safeguarding privacy, and protecting intellectual, time and financial investments by public health staff is often not well regulated or standardized, resulting in protective policies on sharing of public health data in general [64, 65].

### Legal barriers

These barriers are legal instruments used to restrict data sharing, resulting from the underlying willingness (or not) to share data. Solutions to this group of barriers include legal instruments to facilitate data sharing and are highly dependent on solutions to underlying political barriers.

17. *Ownership and copyright.* Agencies that collect public health data are often responsible for the protection of individual and community privacy and may feel that a guardianship or ownership role is bestowed on them by the public [37, 66–68]. This could result in a default of restricting access to most data [37]. Copyright can be used to restrict rather than expand access to data. In practice, it is often not well documented or known who owns public health data, resulting in inconsistent ad-hoc guidelines [37, 62–65, 69]. For example a project in Canada to integrate National Population Health Survey data with provincial data required a different approval process in each province [64].
18. *Protection of privacy.* Public health agencies have the mandate and authority to collect private data from the population governed by the Health Insurance Portability and Accountability Act (HIPAA) in the US or similar legislation in other countries [12, 36, 37, 44, 57, 59, 62, 64, 66–68, 70–73]. A clear distinction between data containing personal identifiers and fully anonymous data may not always be possible, leading to restrictive policies on all types of data due to privacy concerns [12, 36, 37, 74, 75]. Aggregated data without personal identifiers may not be sufficiently detailed for certain applications. Existing tools and standards for the de-identification of personal identifiers such as statistical data masking [19] may not be known or available in many contexts [12, 59].

### Ethical barriers

These are normative barriers involving conflicts between moral principles and values. Solutions for these barriers will involve a global dialogue among all stakeholders on the ethical principles that should govern data sharing.

19. *Lack of proportionality.* The issue of proportionality, the careful deliberation in assessing the risks and benefits that derive from the amount and type of data requested compared to the potential impact of its secondary use, has been identified as a guiding ethical principle for public health data sharing [9]. Public health agencies may disagree with data requestors about the proportional risks and benefits of the secondary use of data and its impact on public health [76].
20. *Lack of reciprocity.* Data sharing practices have not always been fair, and data producers have often felt exploited in transactions where they receive little credit or benefit from their work, while data users that can rapidly analyze data and publish results benefit from academic credit and career advancement [77, 78] as has happened in the past [50–52].

## Discussion

Using a systematic review of evidence from peer-reviewed and non peer-reviewed literature, we identified 20 unique real or potential barriers grouped in a taxonomy of technical, motivational, economic, political, legal, and ethical barriers. The complex interactions between tangible and intangible barriers at different levels can severely limit the effectiveness of isolated solutions. Strategies to resolve specific barriers may not advance data sharing at all if related barriers are not addressed as well in a comprehensive approach or if more fundamental barriers remain unchanged. Specific data sharing strategies should be tailored to different types of data. We focused this review on routinely collected population derived data such as disease surveillance, intervention coverage, or cause specific mortality data. These types of data are widely collected at ever growing spatiotemporal resolution and the extended use of this vast resource for research and policy making could greatly accelerate public health strategies and programs. The effective advancement of data sharing in public health will require a comprehensive understanding of all barriers and a global consensus on the value and on the principles of data sharing.

Most technical, motivational, and economic barriers are deeply embedded in much larger challenges of health information system capacity, particularly in low- and middle income countries. Solutions are being developed as part of major international initiatives including infrastructure development, capacity building, and efficient financing [14, 25, 29, 31, 79]. For example, the need for sustainable financial mechanisms to create capacity and infrastructure for collection and sharing of public health data has been emphasized previously, especially for low income settings [14, 79]. According to Global Fund estimates, 5-10% of program funds should be invested in data collection, monitoring, evaluation and operational research [2]. Global health partnerships and disease specific programs should use ongoing and additional funding to strengthen public health data systems and available data could be used more efficiently through joint use of integrated data for program monitoring and evaluation [2, 3, 5, 6]. The increased use of standards and connected electronic data systems can accelerate the collection and integration across countries of basic longitudinal information such as counts of disease cases and deaths and coverage of interventions. Investments in such routine data systems will better position agencies to address ongoing challenges as well as new public health threats such as the current Ebola crisis in West Africa [80].

Various initiatives have successfully applied solutions for sharing of health data, such as the International Household Survey Network, the Demographic and Health Survey (DHS), the Multi-Indicator Cluster Surveys (MICS), and the International Network for the Continuous Demographic Evaluation of Populations and Their Health (INDEPTH) [2, 81, 82]. The solutions implemented by these initiatives should be translated to routine public health settings.

Political, legal, and ethical barriers will require a different approach. These barriers are less tangible and transparent compared to technical barriers and will need to be clearly outlined and presented for a dialogue across sectors with international agencies such as the World Intellectual Property Organization (WIPO), the World Health Organization (WHO), the World Trade Organization (WTO), countries, development and funding agencies, and experts in ethics and law [83]. This should lead to the creation of a political framework in the form of resolutions or a treaty, and operational guidelines for data sharing in public health [35, 37]. A centralized mechanism such as a commission or secretariat should monitor, mediate, and facilitate data sharing among various stakeholders to ensure a fair and efficient use of data for the advancement of population health.

Most evidence for this review concentrated on disease surveillance or demographic data. More published evidence is needed on sharing of other types of public health data such as genomic data on emerging pathogens or cost data of public health interventions. Although we found the majority of evidence in the peer-reviewed literature (68%), most documents were based on experience or ideas (46%) instead of empirically derived information (22%). Levels of evidence were also different for each barrier. Lack of data collection and metadata and privacy issues were very well documented while no empirical evidence was available for other barriers such as data preservation and format or restrictive policies and data ownership. In-depth formative research is needed to expand the evidence base of these barriers. As knowledge on these barriers will increase, so will opportunities for solutions.

## Conclusion

Great opportunities have been created for global health cooperation, scientific discovery, and effective disease control programs by recent advances in public health data collection. These advancements are contrasted by real and potential barriers that limit the efficient use of these data. A global process will be essential for a more effective use of known solutions and to build consensus for new solutions to harness the potential of data towards a 21st century population health.

## Electronic supplementary material

Additional file 1: **PRISMA checklist.** (PDF 214 KB)

Additional file 2: **Literature search protocol.** (PDF 308 KB)
