# Supplementary material for: A systematic review of barriers to data sharing in public health
Source: BMC Public Health. 2014 Nov 5;14:1144. doi: 10.1186/1471-2458-14-1144 (PMC4239377; doi:10.1186/1471-2458-14-1144)
Supplement: Supplementary file 2 — Additional file 2: Literature search protocol. (PDF 308 KB) [file 12889_2014_7242_MOESM2_ESM.pdf]

SYSTEMATIC REVIEW PROTOCOL: BARRIERS TO SHARING DATA IN PUBLIC HEALTH

Background

The availability and interpretation of data continues to be an integral part of public health decision making. Benefits of data sharing have been widely recognized but in reality, multiple challenges have limited wide-scale sharing of public health data. A variety of potential and real barriers to sharing data have been recognized but so far have only been anecdotally discussed or presented. The current discussion of data sharing has focused primarily on research data and the potential value of population level data routinely collected by public health agencies for science and decision making is poorly recognized and not well characterized in the peer-reviewed literature. Evidence on the scope and variety of challenges that currently limit data sharing in this domain is also limited. Unless these barriers are better understood, the vast opportunities provided by routine public health data would remain under-utilized.

Review objective

To compile published evidence on real and potential barriers to sharing of routinely collected public health data through a systematic review of scientific and related literature and to organize these barriers into a taxonomy that can be used as framework towards solutions.

Inclusion criteria

Types of documents

This review will consider the following types of documents: peer-reviewed articles with empirical data presented, peer-reviewed articles with no/little original data presented, and reports from global health agencies.

Types of data

Reviewed documents have to discuss barriers to sharing of routinely collected public health data. For the purposes of this review public health data is defined as data that is primarily collected by public health agencies for routine purposes such as disease surveillance or program monitoring without primary intention of research. Articles which focus on research, clinical, or other types of data will be excluded.

Types of measures

The review will identify barriers to data sharing. Barriers are defined as obstacles that could hinder or delay the sharing of data or that could limit the efficiency of the sharing process. Like barriers will be grouped together.

Search strategy

This search strategy is designed to access published and unpublished materials through the following steps:

1. MEDLINE search will be limited to English-language articles identified using the combination of two search queries:

   a. *Search terms in query 1:* [“public health” OR “world health”] AND ["data sharing" OR "data access” OR "open access" OR "dissemination" OR "sharing practices"] AND [“barriers” OR “challenges”].
   b. *Search terms in query 2*: [“population surveillance” OR "health statistics" or "vital statistics" or "civil registry" or "health data"] AND ["data sharing" OR "data access” OR "open access" OR "dissemination" OR "sharing practices"] AND [“barriers” OR “challenges”].
2. Bibliographies from retrieved articles will be reviewed and additional articles identified based on title.
3. Public health agency websites (e.g. the World Health Organization, the US Centers for Disease Control, Wellcome Trust, and the Bill & Melinda Gates Foundation) will be searched for relevant reports, based on title.

Two reviewers will independently screen the title and abstract based on the inclusions criteria. Discrepancies between selected articles will be resolved through discussion to reach a consensus.

Data collection

Two reviewers will independently read eligible documents and barriers will be extracted. Both reviewers will be agnostic regarding the types of barriers to look for. Specific barriers will be grouped by domain and related evidence will be reviewed by domain experts among the authors. The final list of barriers will be determined and agreed on by all the authors.

Data synthesis

Individual barriers will be grouped into larger thematic categories to create a taxonomy. A qualitative description of each barrier will be presented in the review per category in the taxonomy. All evidence found for each barrier will be listed grouped by peer-reviewed vs. non peer-reviewed evidence and by empirical vs. non-empirical evidence.
